# Insights Into History and Trends of Teaching and Learning in Stomatology Education: Bibliometric Analysis

**DOI:** 10.2196/66322

**Published:** 2025-10-20

**Authors:** Ziang Zou, Linna Guo

**Affiliations:** 1 Department of Gynecology and Obstetrics, The Third Xiangya Hopital, Central South University, Changsha, Hunan, China; 2 Department of Stomatology, The Second Xiangya Hopital, Central South University, 139 Renmin Middle Road, Changsha, Hunan, 410011, China, 86 18075181927

**Keywords:** teaching, learning, stomatology, education, bibliometric analysis, trend, stomatology education, visualization, education, R-Bibliometrix, CiteSpace, university, innovation, teaching modality, web of science, WOS

## Abstract

**Background:**

Stomatology education has experienced substantial transformations over recent decades. Nevertheless, a comprehensive summary encompassing the entirety of this field remains absent in the literature.

**Objective:**

This study aimed to perform a bibliometric analysis to evaluate the research status, current focus, and emerging trends in this field over the last two decades.

**Methods:**

We retrieved publications concerning teaching and learning in stomatology education from the Web of Science core collection covering the period from 2003 to 2023. Subsequently, we conducted a bibliometric analysis and visualization using R-Bibliometrix and CiteSpace.

**Results:**

In total, 5528 publications focusing on teaching and learning in stomatology education were identified. The annual number of publications in this field has shown a consistent upward trend. The United States and the United Kingdom emerged as the leading contributors to research. Among academic institutions, the University of Iowa produced the highest number of publications. The *Journal of Dental Education* was identified as the journal with the highest citation. Wanchek T authored the most highly cited articles in the field. Emerging research hotspots were characterized by keywords such as “deep learning,” “machine learning,” “online learning,” “virtual reality,” and “convolutional neural network.” The thematic map analysis further revealed that “surgery” and “accuracy” were categorized as emerging themes.

**Conclusions:**

The visualization bibliometric analysis of the literature clearly depicts the current hotspots and emerging topics in stomatology education concerning teaching and learning. The findings are intended to serve as a reference to advance the development of stomatology education research globally.

## Introduction

In recent years, innovations and restructuring in stomatology education have triggered global reforms in the field. In stomatology education, teaching and learning are both interdependent and mutually reinforcing. Effective education demands not only that instructors possess robust professional knowledge and use sound pedagogical strategies but also that students actively participate, provide feedback (FB), and engage in self-directed learning. The dynamic interaction between teaching and learning facilitates the effective transfer and application of knowledge, ultimately resulting in the cultivation of stomatology professionals who are equipped with both advanced technical skills and strong clinical reasoning abilities.

Teaching and learning in stomatology education have undergone profound and meaningful changes in the past two decades. For instance, significant research efforts have been dedicated to using digital and artificial intelligence (AI) tools to enhance the quality of education and exploring the effectiveness and response of online and distance teaching modalities. Hence, it is crucial to understand the latest trends and developments in teaching and learning in stomatology education. However, keeping up with the rapid changes in stomatology education research is challenging. Scientific publications are important for academic communication and clinical practice guidelines. Evaluative bibliometrics, a quantitative science, assesses research performance to identify influential works in medical practice and research. Unlike literature reviews, which offer qualitative insights, bibliometric analysis provides quantitative data on citations, authorship, and research collaboration [1,2]. Although various fields have been studied, teaching and learning in stomatology education research are limited, with few quantitative analyses. Therefore, bibliometric analysis is essential to fill this gap, offering critical data to identify trends and guide future research.

In this study, we conducted a comprehensive bibliometric analysis of teaching and learning in stomatology education using Citespace software (developed by Chaomei Chen, College of Computing and Informatics, Drexel University, Philadelphia, PA, USA) to generate visual representations. The aim was to systematically evaluate the research landscape, current focal areas, and emerging trends in teaching and learning in stomatology education over the past two decades, emphasizing key achievements and outlining potential future research directions in this field.

## Methods

### Search Strategy and Eligibility Criteria

Scholarly articles and literature on teaching and learning in stomatology education were retrieved using the Web of Science Core Collection (WoSCC), encompassing the Science Citation Index Expanded and other relevant citation indices. To ensure the validity and reproducibility of our search strategy, we implemented a multistep validation process. A pilot search was conducted in 2023 using a subset of keywords, yielding 200 randomly selected articles that were manually screened by the authors. This process resulted in a precision rate of 92% based on which minor refinements were made to keyword synonyms. Second, the search strategy was developed in accordance with the PRISMA-ScR (Preferred Reporting Items for Systematic Reviews and Meta-Analyses extension for Scoping Reviews) checklist to ensure methodological rigor and transparency. Third, to assess the comprehensiveness of our chosen database and minimize selection bias, we cross-validated the WoSCC coverage with PubMed and Scopus using a random sample of 100 articles, confirming a 95% overlap in relevant literature. Finally, both controlled vocabulary (MeSH terms) and free-text keywords were used to ensure the search captured a wide range of relevant studies across varying indexing practices. To ensure comprehensive literature retrieval, we designed the search strategy by mirroring MeSH hierarchies and incorporating their synonymous expressions ('Oral medicine’ [Mesh] → dentistry/stomatology); ('Education’ [Mesh] → teaching/training/learning).

The final search string combined controlled vocabulary (MeSH terms) and free-text terms, as follows:

1# :(((TS=(teaching)) OR TS=(training)) OR TS=(learning)) OR TS=(education)

2# :(((TS=(oral medicine)) OR TS=(stomatology)) OR TS=(dentistry))

3# :1# and 2#

The duration of this span is from January 2003 to December 2023. Articles lacking unique content and those published in languages other than English were excluded.

### Ethical Considerations

As the data were directly obtained from the database, ethical approval was not required.

### Bibliometric Analysis

The data from these articles were imported and integrated using the bibliometrix package (version 4.4.0) in the R programming language. Our research applied several bibliometric analysis techniques, including keyword co-occurrence and historical direct citation analyses. For this study, we used CiteSpace (version 6.2R6) to perform social network analysis and to identify developmental dynamics, hotspots, future trends, and key aspects within the scientific literature on a specific topic. Burst detection methodology was applied to pinpoint significant keywords or references that experienced a notable surge in frequency within a defined period. In addition, we conducted a clustering (co-citation) analysis of references using a metric based on co-authored publications, allowing for the grouping of institutions or keywords that showed higher levels of collaboration within the same cluster. Clusters of related publications were generated using CiteSpace’s fully automated cocitation clustering algorithm with the following parameters:

- Clustering method: Louvain modularity optimization (to maximize intracluster connectivity and intercluster distinction)
- Similarity threshold: default cosine similarity (0.5) to balance sensitivity and specificity
- Time slicing: 2-year intervals (2003–2023) to capture temporal trends
- Node type: author keywords+cited references to integrate semantic and bibliometric signals
- Labeling: algorithm-generated labels via Term Frequency-Inverse Document Frequency (TF-IDF )weighting of high-frequency keywords within each cluster.

No manual adjustments were made to cluster labels to avoid subjective bias. Network maps were used to illustrate variations in the quantity or frequency of published records across clusters of similar research topics, with node size and color indicating these differences. The strength of collaborations between nodes was depicted by connecting lines, where thicker lines indicated stronger collaborations. To further enhance data visualization, we used an overlay visualization technique in which the color of each node represented the average year of occurrence for each institution or keyword. The data were saved in plain text format with full record and cited references from WoSCC and then imported into CiteSpace for further analysis.

The bibliometric data for this study were retrieved from Web of Science, and no additional filtering was applied to remove self-citations. However, as none of the authors have previously published related articles in this field, self-citations do not impact the findings of this analysis.

## Results

### Brief Description of Teaching and Learning in Stomatology Education Literature

As of December 31, 2023, a total of 8831 articles were retrieved from the WoSCC database. Of these, 6532 were published between 2003 and 2023, with 6381 articles written in English. The dataset includes 5528 research papers, representing 86.6% of the total, and 593 review articles, constituting 9.3%. The 5 leading WOS disciplines identified are dentistry oral surgery medicine (n=3375), education scientific disciplines (n=844), medicine general internal (n=510), public environmental occupational health (n=500), and health care sciences services (n=413).

### Productivity Analysis

**Figure 1.**
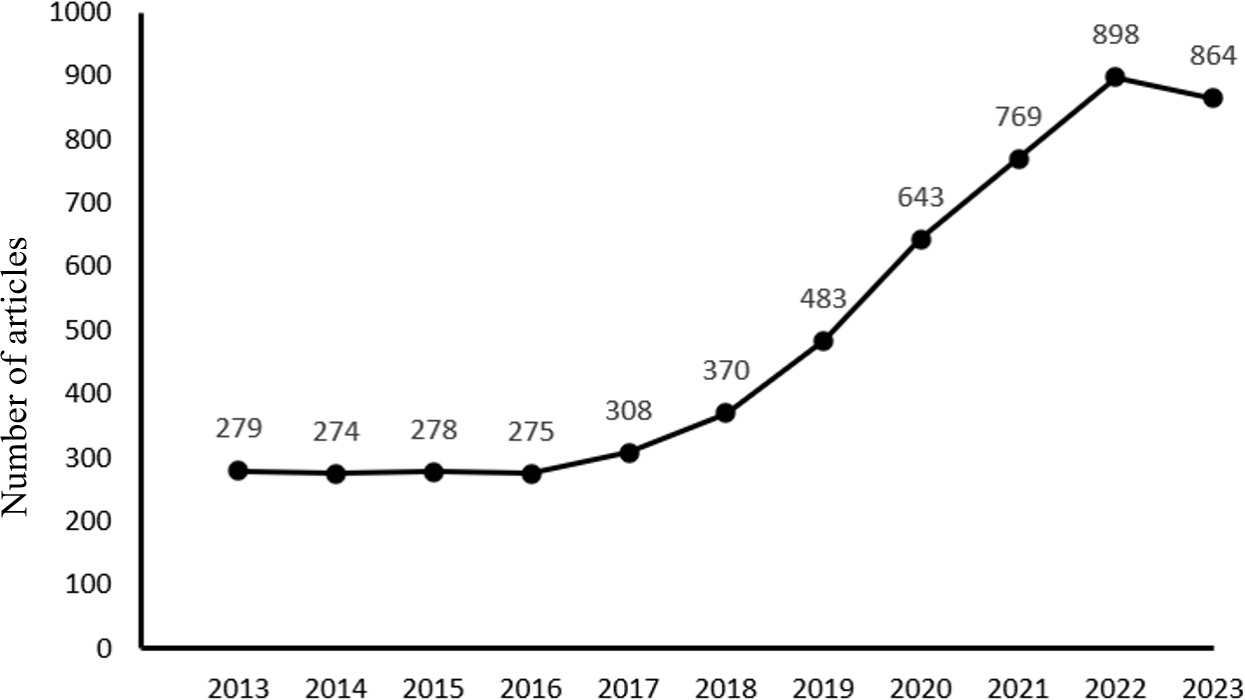
Annual scientific production in teaching and learning of stomatology education.

As demonstrated in 1, the annual publication volume from 2013 to 2023 exhibited distinct temporal patterns. The platform period (2012-2017) maintained stable output with minor fluctuations (270-308 articles per year), followed by an exponential growth phase (2018‐2022) that saw publications surge from 370 to 898 articles per year. Although there was a slight decline in the number of publications from 2022 to 2023, the overall trend remains upward, with the growth rate generally following an exponential pattern. This indicates that international attention to research in stomatology education is steadily increasing and holds promising prospects (Figure 1).

### Countries and Cooperation Networks Analysis

Since 2003, the literature on the research of teaching and learning in stomatology education has been published in 145 countries, mainly led by the following few countries: the United States, the United Kingdom, and China. The number of publications and the frequency of citations are 2 dimensions for analyzing the strength of scientific research, reflecting the attention and impact of a country or institution in a certain field. From the perspective of the number of publications, the top 5 countries are the United States (n=2085), the United Kingdom (n=400), China (n=443), Australia (n=435), and Brazil (n=361). From the perspective of literature citation frequency, the top 5 countries are the United States, the United Kingdom, China, Germany, and Canada. It is worth noting that the United States, the United Kingdom, and China all rank among the top 3 in terms of the number of publications and the frequency of citations, which proves that these 3 countries have strong scientific research capabilities in this field (Table 1).

**Table 1.** Top 10 productive countries concerning teaching and learning in stomatology education.

Country	Documents, n (%)	Citations	Average citations	Centrality
The United States	2085 (37.7)	25,599	16.00	0.24
The United Kingdom	800 (14.5)	7942	14.10	0.13
China	443 (8.0)	6125	15.30	0.04
Germany	310 (5.6)	4405	20.10	0.02
Canada	348 (6.3)	3053	14.30	0.02
Australia	435 (7.9)	3023	12.80	0.11
Brazil	361 (6.5)	2756	11.10	0.03
Turkey	161 (2.9)	2387	16.80	0.00

**Figure 2.**
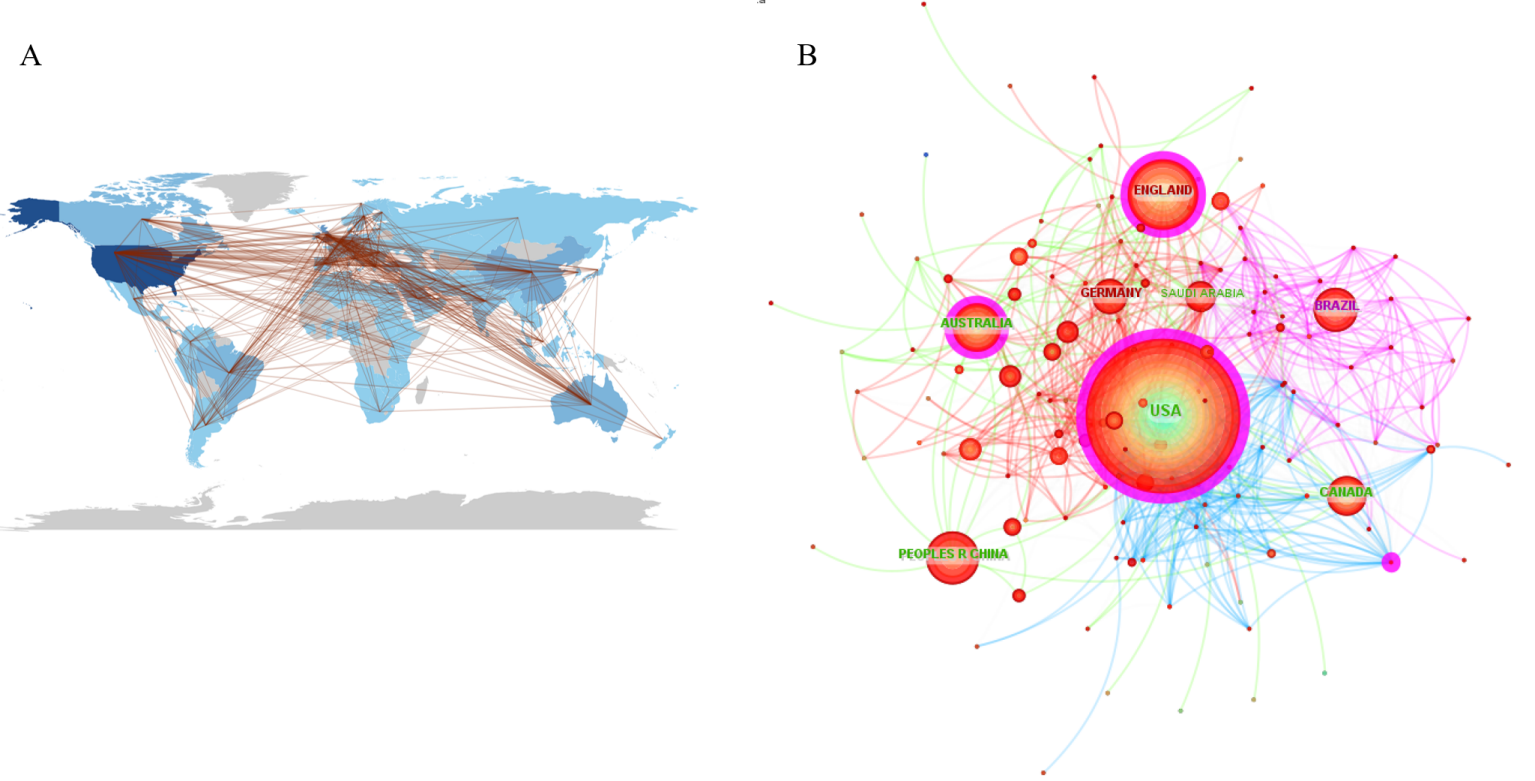
Leading countries in teaching and learning of stomatology education. (A) Countries’ collaboration world map. (B) Countries’ cooperation network.

The data were imported into CiteSpace for a cooperation network analysis, where the centrality of each country was calculated. As illustrated in Figure 2B and detailed in Table 1, each circle represents a country; a darker center color indicates earlier publication dates. Higher centrality values reflect stronger collaboration with other nations. Circles with purple outer rings signify particularly high centrality. Research on teaching and learning in stomatology education began earlier in the United States, the United Kingdom, and Australia. The United States exhibits the highest centrality (0.20), followed by the United Kingdom (0.13) and Australia (0.11), suggesting these countries prioritize international collaboration in this field. Notably, while China (0.04), Germany (0.02), Canada (0.02), Brazil (0.03), and Turkey (0.00) rank among the top 10 in terms of publication and citation counts, their centrality is significantly lower than that of the United States, the United Kingdom, and Australia (Table 1). “Centrality” reflects a country’s role as a hub in global research collaboration. A higher centrality value (eg, the United States with 0.20) indicates stronger connectivity and influence in bridging diverse research communities. Therefore, the United States served as a central node connecting European and Asian institutions in stomatology education research. The national cooperation network analysis reveals that the connections between these countries and others are relatively robust, indicating extensive international cooperation (Figure 2).

### Institutions and Cooperation Analysis

**Figure 3.**
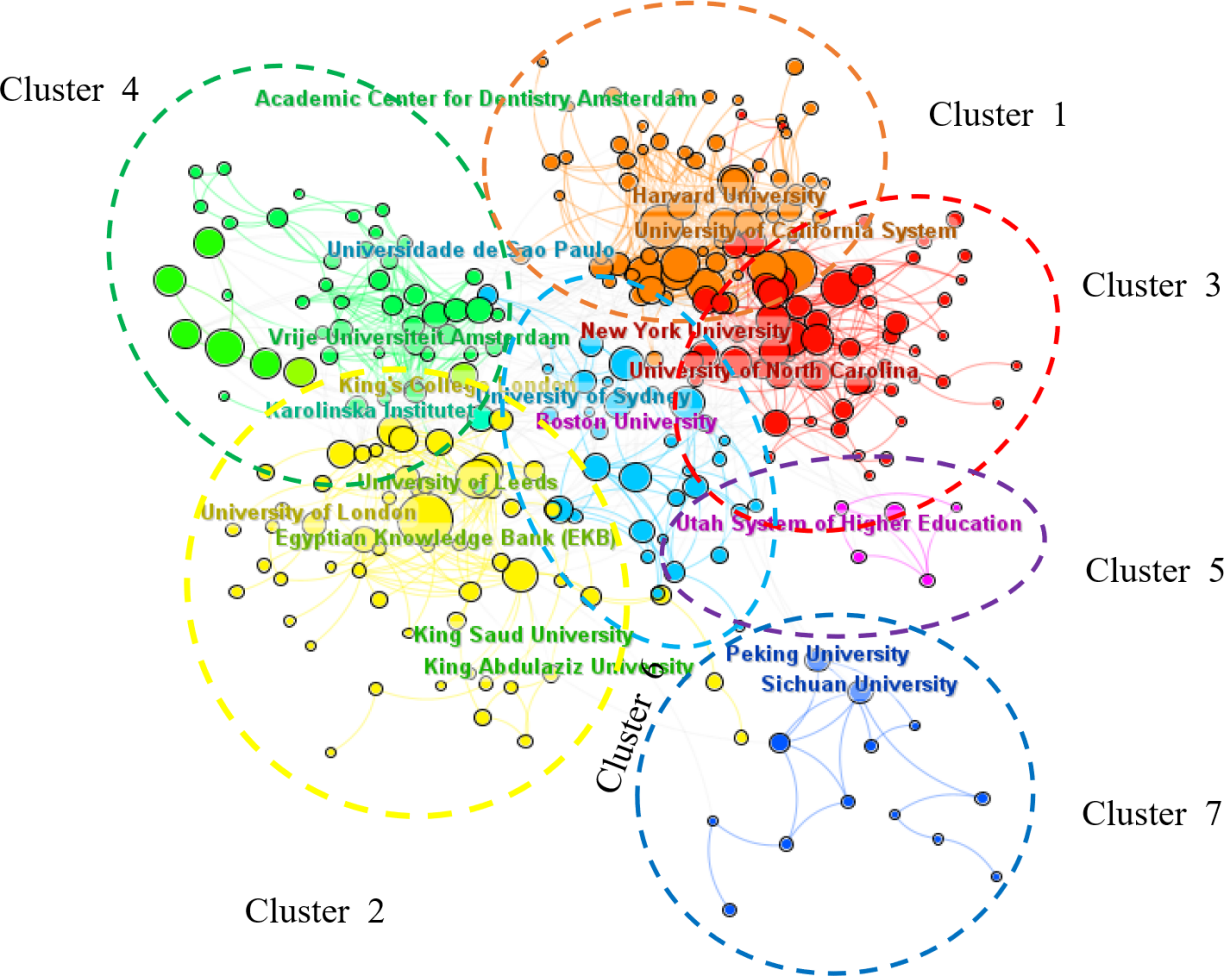
Visualization of active institutes and cluster analysis of cooperation among institutes.

As presented in Table 2, the top 10 institutions in teaching and learning in stomatology education–related research are listed. The top 3 institutions are all from the United States, namely UNIV IOWA (n=305), UNIV MICHIGAN (n=288), UNIV N CAROLINA (n=257, Table 2), while KINGS COLL LONDON (n=236) from the United Kingdom, UNIV SYDNEY from Australia (n=155), and KING SAUD UNIV (n=160) and UNIV HONG KONG (n=153) from Asia have also made outstanding contributions. A comprehensive analysis of cooperative networks among institutions or universities was conducted. In the cooperative network analysis chart, a larger font size signifies that the institution not only began relevant research earlier but has also made more significant contributions to the field (Figure 3). As shown in Figure 3, the University of London, the University of California, King’s College London, Boston University, Harvard University, and Peking University have played an important role in the development of this field. In the network map, cluster analysis of cooperation among institutes and overlay visualization of the largest 7 clusters was shown in Figure 3. The top-ranked item by centrality is the National and Kapodistrian University of Athens (0.14, cluster 2). The second one is the Cleveland Clinic (0.09, cluster 6). The third is the Case Western Reserve University (0.08, cluster 6). The fourth is the University of Sheffield (0.07, cluster 4). The fifth is the Pennsylvania Commonwealth System of Higher Education (0.07, cluster 1).

**Table 2.** Top 10 productive institutions concerning teaching and learning in stomatology education.

Affiliation	Country	Publication counts
UNIV IOWA	USA	305
UNIV MICHIGAN	USA	288
UNIV N CAROLINA	USA	257
KINGS COLL LONDON	The United Kingdom	236
UNIV ILLINOIS	USA	185
KING SAUD UNIV	Saudi Arabia	160
UNIV SYDNEY	Australia	155
UNIV HONG KONG	China	153
UNIV WASHINGTON	USA	153

### Authors’ Analysis

We conducted an analysis of the authors of the published articles and identified the top 5 authors by citation count: Wanchek T (n=132), Cook BJ (n=123), Valachovic RW (n=114), Mattheos N (n=91), and Lynch CD (n=91). The affiliations of these authors, including their respective countries and institutions, are detailed in Table 3. Notably, 3 of these authors are affiliated with the University of Virginia, the United States (Table 3).

**Table 3.** Top 10 most productive authors in teaching and learning in stomatology education.

Author	Country	Institution	Citations
Wanchek T	USA	University of Virginia	132
Cook BJ	USA	University of Virginia	123
Valachovic RW	USA	University of Virginia	114
Mattheos N	Sweden	Karolinska Institute	91
Lynch CD	Ireland	University Dental School and Hospital Wilton	88
Kalenderian E	USA	Harvard School of Dental Medicine	72
Anderson EL	USA	American Dental Education Association	71
Lee JY	South Korea	Wonkwang University College of Dentistry	70
Divaris K	USA	University of North Carolina	63
Wilson NHF	The United Kingdom	King’s College London Dental Institute	63

**Figure 4.**
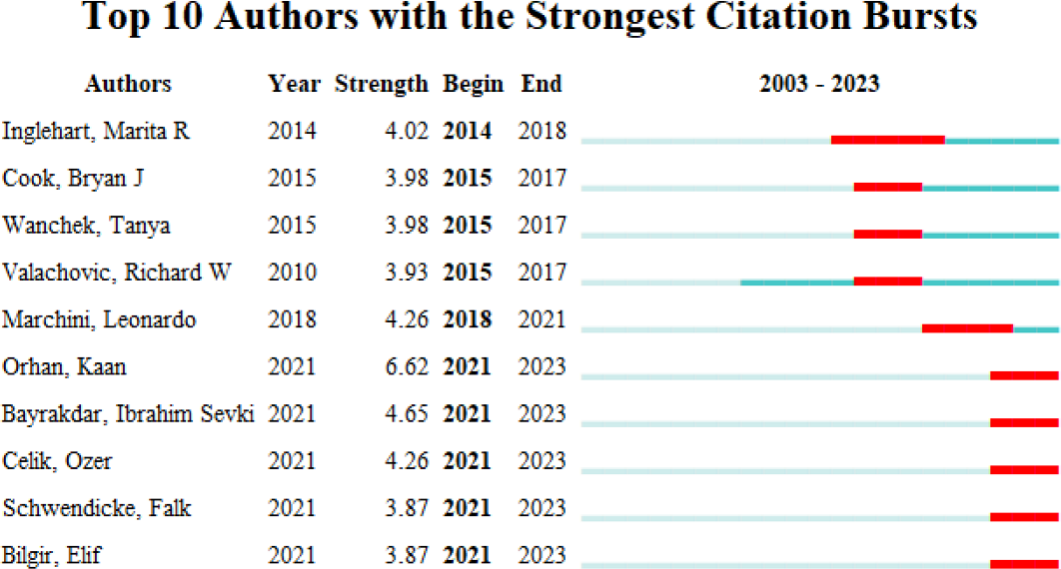
Top 10 authors with the strongest citation bursts.

Using the “urst” function, we identified the most active authors in the field, as illustrated in Figure 4. Analysis of the burst detection results indicates that teaching and learning in stomatology gained prominence starting in 2014, with significant contributions from Inglehart MR (USA). During 2021 to 2023, Orhan K emerged as the most active author in the field, achieving the highest burst strength of 6.62. Bayrakdar IS, with a burst strength of 4.65, ranked second and had an active period from 2021 to 2023. Consequently, Orhan K and Bayrakdar IS not only demonstrated strongest citation bursts but also positioned themselves as a leading researcher in recent years. In addition, Celik O, Schwendicke F, and Bilgir E have recently held prominent positions in this research frontier (Figure 4).

### Journal Analysis

The top 10 journals with the highest citation counts are presented in the table below. The 5 most cited journals include the *Journal of Dental Education* (n=9120), *European Journal of Dental Education* (n=4963), *Journal of Dental Research* (n=2679), *British Dental Journal* (n=2618), and *Journal of the American Dental Association* (n=2238). Impact factors (averaged over the past 5 years) and Journal Citation Reports (JCR) are detailed in Table 4. When examining the H-index of the cited articles, the leading 5 journals are *Journal of Dental Education* (H=36), *European Journal of Dental Education* (H=31), *British Dental Journal* (H=25), *Journal of the American Dental Association* (H=25), and *BMC Oral Health* (H=23), as presented in Table 4. Consequently, the *Journal of Dental Education* and the *European Journal of Dental Education* have been particularly influential, with a strong focus on research related to teaching and learning in dental education.

**Table 4.** Top 10 core journals on teaching and learning in stomatology education.

Journal	H_index	Counts	Citations	IF^a^	JCR^b^
*Journal of Dental Education*	36	841	9120	1.7	Q3
*European Journal of Dental Education*	31	444	4963	2.2	Q2
*British Dental Journal*	25	267	2618	2.1	Q2
*Journal of the American Dental Association*	25	87	2238	3.4	Q1
*BMC Oral Health*	23	129	1613	3.2	Q1
*Journal of Dental Research*	21	50	2679	5.7	Q1
*Journal of Dentistry*	20	55	1511	5	Q1
*Academic Medicine*	17	29	699	6.7	Q1
*Community Dentistry and Oral Epidemiology*	17	50	822	2.7	Q2
*International Journal of Paediatric Dentistry*	17	38	770	2.9	Q2

a IF: impact factors.

b JCR: Journal Citation Reports.

### Research Focus and Frontiers Analyses on the Nonthermal Plasma Medicine

#### Research Focus Analysis

**Figure 5.**
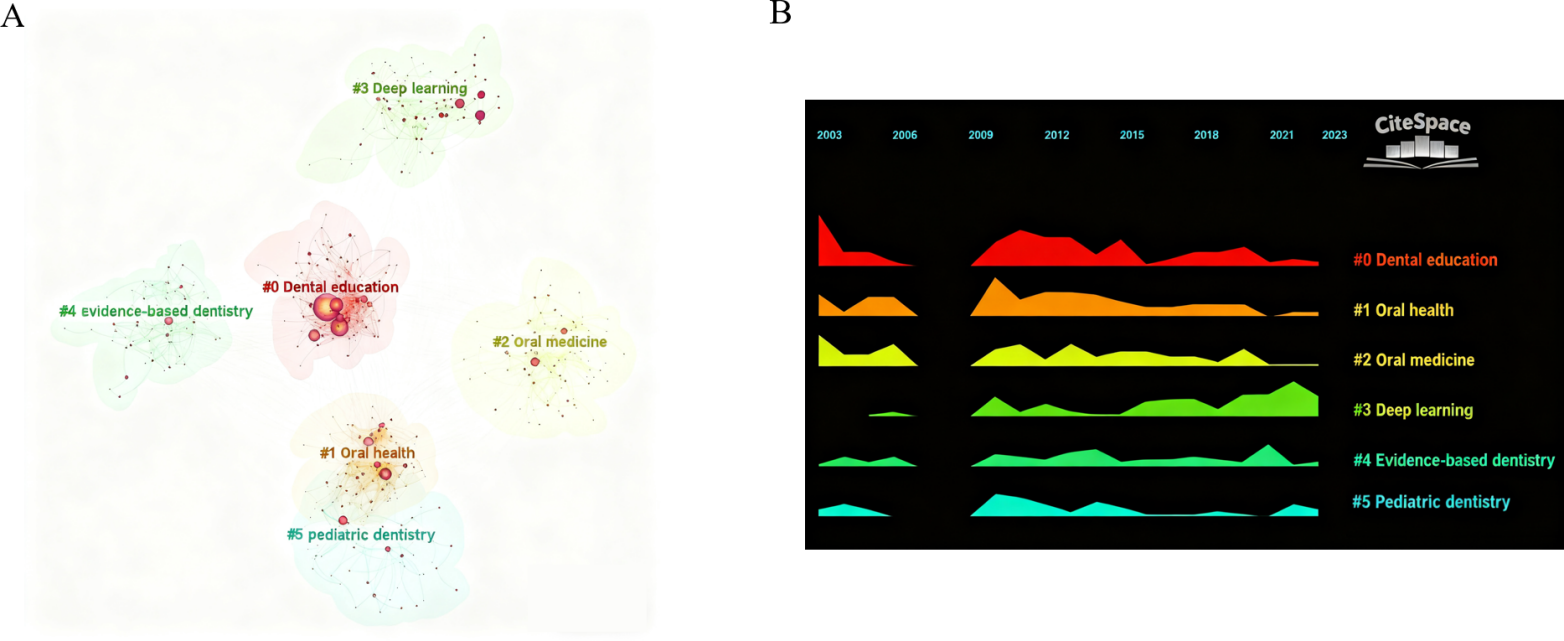
Visualization of keywords analysis. (A) Keywords co-occurrence and centrality. (B) Timeline of keywords co-occurrence.

The word frequency and centrality of keywords reflect the hot topics that researchers have focused on and studied over a period. Keywords mark the core meaning of an article, and conducting keyword analysis can help deeply grasp the core content of this field. In the topic and keyword co-occurrence analysis, nodes reflect the frequency of keywords. Centrality is a basic indicator to measure the weight of nodes, reflecting the importance of nodes in the network. The more prominent the co-occurrence frequency and centrality of keywords, the stronger the importance of nodes. The keyword co-occurrence analysis is shown in Figure 5A. Node size reflected the frequency of keywords. In Figure 5A, the nodes with purple outer circles were those with higher centrality, among which “disease” (0.10), “access” (0.07), “performance” (0.06), “diagnosis” (0.06), and “model” (0.06) possessed higher centrality and frequency, indicating these key contents that have been paid attention to in the promotion and development process of the field (Table 5). Meanwhile, through further sorting and summarizing high-frequency keywords, it was found that the research hotspots in the field of nonthermal plasma medicine from 2003 to 2023 were mainly concentrated in 4 categories. The keyword with the highest level of attention in cluster 0 is “dental education,” the keyword with the highest level of attention in cluster 1 is “oral health,” the keyword with the highest level of attention in cluster 2 is “prevalence,” the keyword with the highest level of attention in cluster 3 is “artificial intelligence,” the keyword with the highest level of attention in cluster 4 is “knowledge,” and the keyword with the highest level of attention in cluster 5 is “impact” and (Figure 5A). Through a timeline analysis of keywords, a chronological map of dental education research was generated. Keywords belonging to the same cluster are aligned horizontally, with their corresponding periods displayed at the top. In this timeline, the density of keywords indicates the significance of the respective clustering domain. Over the past 3 years, “deep learning” has emerged as a prominent research focus in the field of dental education (Figure 5B).

**Table 5.** Top 10 highest centrality keywords.

Keywords	Centrality	Counts
Disease	0.10	74
Access	0.07	58
Performance	0.06	90
Diagnosis	0.06	104
Model	0.06	75
Management	0.05	186
Dentists	0.05	126
Quality	0.05	83
Teeth	0.04	67
Adolescents	0.05	59

#### Research Frontiers Analysis

**Figure 6.**
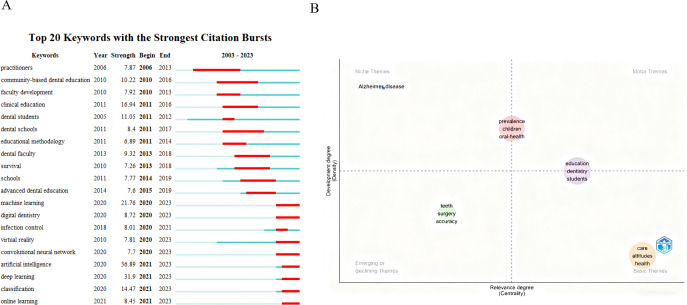
(A) Keywords with the strongest citation burst. (B) Thematic map of keywords in teaching and learning of stomatology education.

Burst detection identifies keywords or authors that experienced sudden surges in citations over a short period. Keywords with the strongest citation bursts mean that the keywords appear more frequently or are cited more frequently in a relatively short period. The detection indicators of keyword citation bursts generally include intensity and age distribution, which reflect the forefront and development trend of research over a period. Figure 6A lists the top 20 keywords with the strongest citation bursts. “Practitioners” appeared the earliest and lasted the longest (2006‐2013), with a strength of 7.87. During this period, “faculty development” and “dental students” are also keywords with high attention. Subsequently, “community-based dental education,” “clinical education,” “dental school,” “educational methodology,” and “advanced dental education” began to become a research hotspot for many scholars after 2011 and lasted until 2019. During this period, “clinical education” (2011‐2016, 16.94), “dental students” (2011‐2012, 11.05), and "community-based dental education" (2010‐2016, 10.22) all received more attention. In the past 4 years, “artificial intelligence” showed the highest burst strength (36.89) from 2020 to 2023, signaling its rapid rise as a cutting-edge topic in teaching and learning of stomatology education. In addition, “deep learning,” “machine learning,” “classification,” “digital dentistry,” “online learning,” “infection control,” “virtual reality,” and “convolutional neural network” have also received much attention from scholars in the past 4 years.

This article uses the Bibliometrix package in the R language environment to describe the history of topic evolution based on keywords (Thematic Map). The results show that “care,” “attitudes,” and “knowledge” were in the “basic themes” area, indicating that these are basic concepts for research in this field. In Figure 6B, the analysis revealed that “teeth,” “surgery,” and “accuracy” were classified as “emerging themes,” indicating that researchers are relatively active in these areas, which may have good prospects in the field of teaching and learning in stomatology education.

### Co-Cited References Analysis

The historiographic map, proposed by E. Garfield in 2004, graphically represents a chronological network of key direct citations from a bibliographic collection. It illustrates the diachronic evolution of highly cited documents, highlighting shifts in research hotspots. Bibliometrix’s historical citation analysis offers 2 metrics: the local citation score (LCS) for citations within the current database and the global citation score (GCS) for total citations in the Web of Science database. LCS counts how often a paper is cited by other studies in the same field, whereas GCS tracks total citations across all disciplines. These metrics assess the impact of documents and reveal connections across research fields. Use the following command to generate the historical citation network: [options (width=130)] [histResults<- histNetwork (M, min. citations=2, sep=“;")] [net<- histPlot (histResults, n=10, size=5, labelsize=3)].

**Figure 7.**
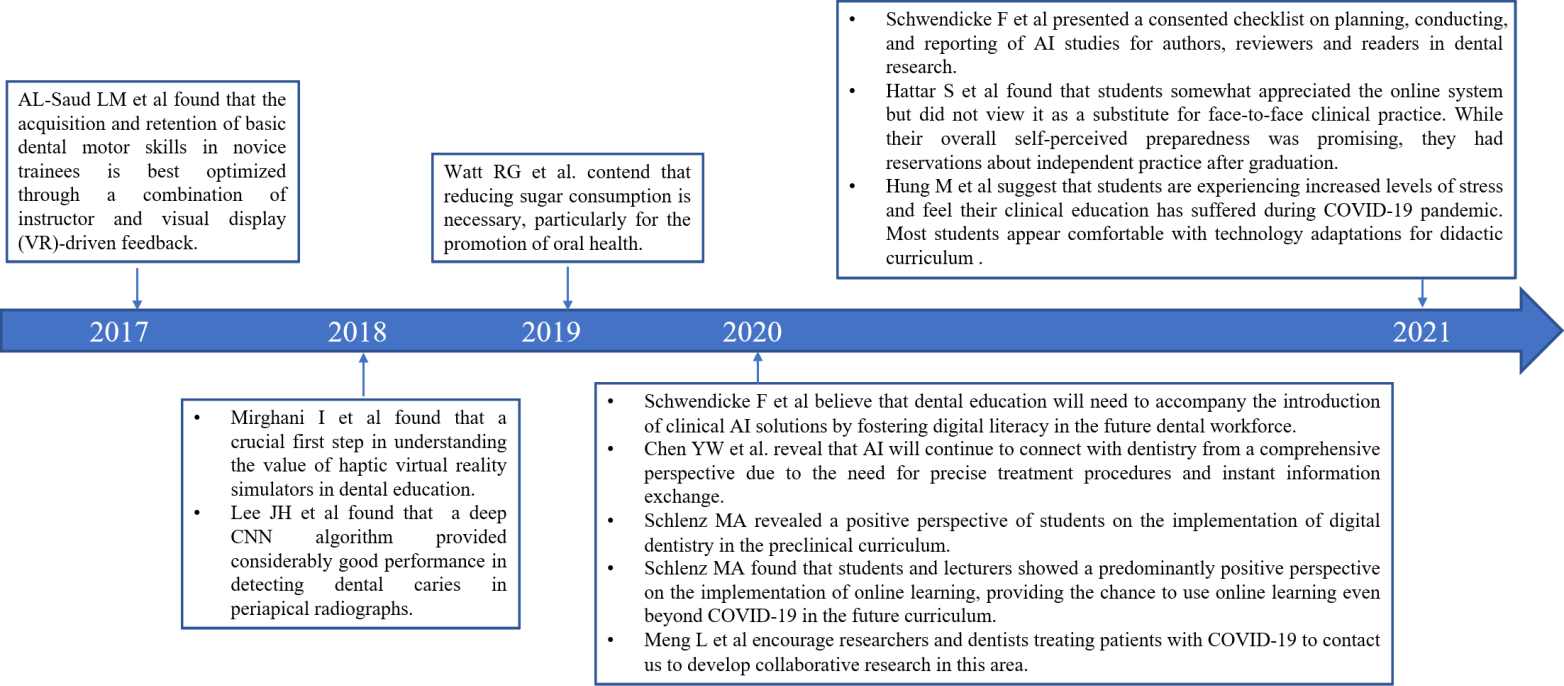
Timeline of part of landmark achievements in teaching and learning of stomatology education. AI: artificial intelligence [5-15].

The analysis results of the historical direct citation network indicated that there were 11 landmark documents in this field, and Table 6 and Figure 7 summarize the relevant information of important documents in the historical cited network atlas. According to the statistics of the 4 articles labeled 4, 5, 8, and 9, the LCS and GCS indices are relatively high, indicating that these 4 articles have a significant impact in the corresponding years, and the research content is highly correlated with the relevant research about teaching and learning in stomatology education.

**Table 6.** Eleven landmark documents in teaching and learning of stomatology education.

Code	Title	Author	Journal	Country	Year	LCS^a^	GCS^b^
1	Feedback and motor skill acquisition using a haptic dental simulator	Al-Saud et al [3]	Eur J Dent Educ	Denmark	2017	24	67
2	Capturing differences in dental training using a virtual reality simulator	Mirghani et al [4]	Eur J Dent Educ	Denmark	2018	19	52
3	Detection and diagnosis of dental caries using a deep learning-based convolutional neural network algorithm	Lee et al [5]	J Dent	The United Kingdom	2018	51	367
4	Ending the neglect of global oral health: time for radical action	Watt et al [6]	Lancet	The United Kingdom	2019	20	427
5	Artificial intelligence in dentistry: chances and challenges	Schwendicke et al [7]	J Dent Res	USA	2020	40	285
6	Artificial intelligence in dentistry: current applications and future perspectives	Chen et al [8]	Quintessence Int	USA	2020	23	99
7	Artificial intelligence in dental research: checklist for authors, reviewers, readers	Schwendicke et al [9]	J Dent	The United Kingdom	2021	16	136
8	Students’ and lecturers on the implementation of online learning in dental education due to SARS-COV-2 (COVID-19): a cross-sectional study	Schlenz et al [10]	BMC Med Educ	The United Kingdom	2020	39	128
9	Coronavirus disease 2019 (COVID-19): emerging and future challenges for dental and oral medicine	Meng et al [11]	J Dent Res	USA	2020	67	914
10	Impact of COVID-19 pandemic on dental education: online experience and practice expectations among dental students at the University of Jordan	Hattar et al [12]	BMC Med Educ	The United Kingdom	2021	25	68
11	In an era of uncertainty: impact of COVID-19 on dental education	Hung et al [13]	J Dent Educ	Denmark	2021	35	101

a LCS: local citation score.

b GCS: global citation score.

## Discussion

### General Information

This study uses bibliometric analysis to examine the key areas of knowledge and emerging trends in stomatology education. Notably, several breakthrough achievements have been identified through these bibliometric methods. The findings reveal a general upward trend in annual publications related to teaching and learning in stomatology education. Furthermore, the geographic distribution of these publications was analyzed based on the country and institutional affiliations of the authors. The results indicate that institutions from the United States, the United Kingdom, China, and Australia are dominant in this field. Specifically, the University of Iowa, the University of Michigan, and the University of North Carolina in the United States; King’s College London in Europe; the University of Sydney in Australia; and King Saud University and the University of Hong Kong in Asia have played pivotal roles in the development of stomatology education. In terms of productivity and influence, Wanchek T from the United States has been at the forefront over the past two decades. His focus on how education debt affects career choices among dental students and professionals has earned him numerous citations [14,15]. The primary objective of this study is not to provide exhaustive precision data on teaching and learning in stomatology education but to stimulate interest and encourage further research in this domain. This article also serves as a valuable resource for researchers new to the field, offering a concise overview of current trends and developments in stomatology education.

The observed increase in publications within this field can likely be attributed to the emergence of specialized journals in stomatology education and the growing interest among researchers in the professional and academic evolution of teaching methodologies. Among the top 10 journals, the Journal of Dental Education and the European Journal of Dental Education stand out, having published the highest number of articles and achieving the highest H-index. These journals have become highly esteemed among researchers worldwide, as they highlight the latest advancements and significant discoveries in stomatology education. In recent years, these journals have particularly focused on the application of AI and its potential impact on stomatology, as well as the role of online learning during the COVID-19 pandemic. These topics align with the findings from keyword bursts, where terms such as “artificial intelligence,” “deep learning,” “machine learning,” “classification,” “digital dentistry,” “online learning,” and “infection control” have emerged as active themes in the field. It is worth mentioning that “artificial intelligence” showed the highest burst strength (36.89) from 2020 to 2023, signaling its explosive growth as a game-changing tool in teaching and learning dental educational skills.

### Current Research Focus

In the current research related to stomatology education, the relatively advanced landmark literature was the article published by Al-Saud et al [3] in 2017, which investigated the effect of qualitatively different types of pedagogical FB on the training, transfer, and retention of basic manual dexterity dental skills using a virtual reality (VR) haptic dental simulator. It is reported that the acquisition and retention of basic dental motor skills in novice trainees is best optimized through a combination of instructor and VR-driven FB. Subsequently, Mirghani et al [4] further emphasized the value of haptic VR simulators in dental education. From here on, virtual teaching was paid attention to dental education, and its FB in teaching and learning was investigated by dental educators.

In the realm of teaching and learning in stomatology education, virtual and AI-based instructional methods serve as complementary approaches. Their integration has the potential to substantially enhance educational outcomes. In 2018, Lee et al [5] demonstrated the potential of a deep learning–based convolutional neural network algorithm for the detection and diagnosis of dental caries. This study marked a significant advancement in the application of deep learning technologies within the domain of dental education, garnering substantial attention. Following this, Watt et al [6] highlighted the potential benefits of integrating deep learning and other intelligent systems into dental education, such as personalized learning experiences and predictive analytics. Nonetheless, they cautioned that the adoption of these technologies must be accompanied by ethical considerations to ensure that they augment rather than replace human judgment in educational contexts. Consequently, deep learning, as a facet of AI, is progressively transforming traditional oral medicine education through enhanced image analysis, virtual simulations, personalized learning, and intelligent systems. The incorporation of AI-driven coaching and doctor–patient communication training has notably improved both the quality and efficiency of dental education. In 2020, Schwendicke et al [7] and Chen et al [8] further explored the integration of AI within dentistry. They asserted that future dental education needs to advance alongside the introduction of clinical AI solutions by cultivating digital literacy among emerging dental professionals. They also emphasized that AI will increasingly interface with dentistry from a holistic perspective, driven by the demand for precise treatment procedures and immediate information exchange.

The COVID-19 pandemic has profoundly impacted dental education by accelerating the adoption of digital and remote learning modalities. With physical distancing measures and health concerns limiting in-person interactions, institutions have increasingly turned to virtual platforms and online resources to ensure continuity of education. This shift has highlighted the necessity for adaptable and resilient teaching methods in oral medicine, fostering the development of new pedagogical strategies and technologies. This has also stimulated a large number of studies, including landmark research by Schlenz et al [9,10], who found that students and lecturers have positive attitudes toward the implementation of online learning. Meng et al [11] also encouraged researchers and dentists treating COVID-19 patients to connect with each other online to carry out collaborative research in this field. The pandemic has underscored the significance of integrating digital tools into the curriculum, which not only addresses immediate challenges but also offers long-term benefits in creating more flexible and accessible educational environments. However, experts believe that there are also some potential problems with online teaching or distance learning. Hung et al [13] suggested that students are experiencing increased levels of stress and feel their clinical education has suffered during the COVID-19 pandemic. Hattar et al [12] found that students somewhat appreciated the online system but did not view it as a substitute for face-to-face clinical practice. While their overall self-perceived preparedness was promising, they had reservations about independent practice after graduation. Lee et al [16] found that in the realm of continuing education in oral and maxillofacial radiology for dentists, the online interactive education emerges as an effective tool, fostering positive academic achievements through active learning.

### Future Frontiers

The future of teaching and learning in stomatology education will be shaped by advancements in AI, digital simulations, and machine learning strategies. Our bibliometric analysis highlights key emerging trends, including deep learning, convolutional neural networks, VR, and surgical accuracy, which are driving innovation in dental education. These developments indicate a growing emphasis on improving diagnostic accuracy, enhancing clinical training, and integrating AI-driven technologies into educational curricula.

One notable trend is the increasing application of VR and AI-assisted simulations in surgical education. Virtual surgical planning and real-time FB systems allow students to rehearse complex procedures in a risk-free environment, thereby improving both skill acquisition and patient outcomes [17]. Similarly, robotic surgery, which offers enhanced precision and control, is gaining traction in advanced dental training [18]. These findings align with our bibliometric results, which identified “surgery” and “accuracy” as emerging themes. To translate these insights into practice, educators should prioritize competency-based training models that integrate VR-based surgical practice and AI-assisted procedural assessments [19]. For example, Huang et al [20] demonstrated that repeated VR training significantly enhanced students’ implantology skills, with positive FB from participants highlighting the technology’s effectiveness. Al-Saud et al [3] demonstrated that haptic dental simulators significantly enhance motor skill acquisition, whereas Mirghani et al highlighted their role in replicating real-world dental procedures, improving student performance and confidence [4]. A study on an inferior alveolar nerve block simulator also found that students who practiced with the simulator were more confident during their first clinical injections, required fewer syringe adjustments, and achieved greater success in numbing patients [21]. These findings highlight the effectiveness of technology-enhanced learning, supporting our bibliometric analysis that identified “VR” and “AI” as emerging trends in stomatology education. Moving forward, further research should explore the long-term impact of these technologies on clinical proficiency and patient care outcomes.

Another key area of transformation is AI-driven diagnostic training. The use of machine learning models to analyze dental imaging data has significantly improved diagnostic precision in fields such as pediatric dentistry and prosthodontics [22]. AI-powered convolutional neural networks have demonstrated high accuracy in detecting caries and occlusal abnormalities [23]. Moreover, the use of AI in dental education is advancing, with studies such as Chen et al discussing its applications in clinical decision-making and personalized training programs [8]. Given this trend, educators should integrate AI-based analysis into preclinical coursework, allowing students to develop advanced diagnostic skills before entering clinical practice.

While AI-driven learning systems enhance efficiency and accessibility, their implementation also raises ethical considerations. Existing research on AI in dentistry exhibits notable deficiencies, impeding its reproducibility and practical implementation. AI should complement, rather than replace, traditional instructor–student interactions [9]. Future research should focus on ethical frameworks for AI integration in education, addressing issues such as student autonomy, decision-making processes, and the potential bias in AI-generated FB. In addition, the interdisciplinary nature of AI-driven education requires stronger collaboration between dental educators, AI developers, and clinical practitioners. Research efforts should focus on optimizing AI algorithms for educational use, ensuring that these technologies align with the pedagogical needs of dental students. By fostering such collaborations, the field can move toward more effective and human-centered educational frameworks.

By aligning bibliometric insights with actionable strategies, educators and researchers can better navigate the evolving landscape of stomatology education. Bibliometric analysis provides a data-driven foundation for curriculum innovation and policy development by identifying emerging trends, evaluating influential studies, and uncovering knowledge gaps. For educators, it helps optimize curriculum design by integrating AI-driven diagnostics, competency-based learning, and virtual simulation training. For researchers, it serves as a strategic tool for identifying high-impact studies, fostering interdisciplinary collaborations, and addressing underexplored areas such as AI ethics and hybrid education models. By leveraging bibliometric methods, stakeholders can make informed decisions that enhance both teaching quality and learning outcomes, ensuring that technological advancements are effectively integrated into stomatology education.

### Limitations and Strengths

This study possesses several notable strengths. Primarily, it represents the first bibliometric analysis of publications on teaching and learning in stomatology education using both the Bibliometrix package and CiteSpace. This analysis offers clinicians and scholars a comprehensive overview of the current landscape in stomatology education. In addition, the simultaneous use of 2 bibliometric tools, CiteSpace and Bibliometrix, enhances the robustness of our findings. CiteSpace, in particular, is a widely recognized tool that provides valuable insights into evolving research priorities and trends.

However, this study also has certain limitations. First, the search strategy was specifically focused on teaching and learning in stomatology education, which, despite efforts to expand the search terms, may have resulted in the exclusion of some relevant studies. Second, our study primarily uses bibliometric methods, which focus on citation patterns, co-occurrence networks, and research trends but do not directly assess the pedagogical effectiveness of emerging technologies. While our findings highlight the increasing focus on machine learning, VR, and AI-driven education, further empirical research is needed to evaluate how these innovations impact student learning outcomes, clinical decision-making, and practical skill acquisition. Conducting comparative studies between traditional and technology-enhanced teaching methods could provide deeper insights into their educational value. In addition, our analysis relies on data from WoSCC, which may exclude relevant publications indexed in other databases such as Scopus or PubMed. This could result in a partial representation of the research landscape. Future studies could incorporate multiple databases to ensure a more comprehensive assessment of publication trends. Finally, emerging research areas related to teaching and learning in stomatology education may have been inadvertently overlooked due to the constraints of the used algorithms.

### Conclusions

In conclusion, the exploration of innovative teaching and learning methodologies holds significant potential for advancing stomatology education. For example, the integration of deep learning models can enhance diagnostic precision and clinical practice, whereas intelligent teaching systems can facilitate more efficient acquisition of oral medicine knowledge and improve educational outcomes. These technological advancements present new challenges for educators in the field. It is imperative that educators remain informed about the latest research developments and evolving trends in stomatology education. This awareness will not only aid in the dissemination of contemporary concepts but also enable the timely identification of emerging issues, thereby fostering the ongoing development and advancement of stomatology education.

## Abbreviations

AI: artificial intelligence
FB: feedback
GCS: global citation score
LCS: local citation score
PRISMA-ScR: Preferred Reporting Items for Systematic Reviews and Meta-Analyses extension for Scoping Reviews
VR: virtual reality
WoSCC: Web of Science Core Collection
